# Interventions to Promote a Healthy Sexuality among School Adolescents: A Scoping Review

**DOI:** 10.3390/jpm11111155

**Published:** 2021-11-07

**Authors:** Fernanda Loureiro, Margarida Ferreira, Paula Sarreira-de-Oliveira, Vanessa Antunes

**Affiliations:** Centro de Investigação Interdisciplinar Egas Moniz (CiiEM), Escola Superior de Saúde Egas Moniz, 2829-511 Almada, Portugal; mferreira@egasmoniz.edu.pt (M.F.); psarreira@egasmoniz.edu.pt (P.S.-d.-O.); vantunes@egasmoniz.edu.pt (V.A.)

**Keywords:** adolescent, nursing, review literature, sexuality

## Abstract

Schools are particularly suitable contexts for the implementation of interventions focused on adolescent sexual behavior. Sexual education and promotion have a multidisciplinary nature. Nurses’ role and the spectrum of the carried-out interventions is not clear. We aimed to identify interventions that promote a healthy sexuality among school adolescents. Our review followed the Preferred Reporting Items for Systematic Reviews and Meta-Analyses extension for Scoping Reviews and was registered in the Open Science Framework. Published articles on sexuality in adolescents in school contexts were considered. The research limitations included primary studies; access in full text in English, Spanish, or Portuguese; and no data publication limitation. Research was carried out on the EBSCOhost, PubMed, SciELO, and Web of Science platforms; gray literature and the bibliographies of selected articles were also searched. A total of 56 studies were included in the sample. The studies used a broad range of research methods, and 10 types of interventions were identified. Multi-interventional programs and socio-emotional interventions showed a greater impact on long-term behavioral changes, and continuity seemed to be a key factor. Long-term studies are needed to reach a consensus on the effectiveness of interventions. Nurses’ particular role on the multidisciplinary teams was found to be a gap in the research, and must be further explored.

## 1. Introduction

The World Health Organization [1] considers sexuality as a central aspect of human beings. It is influenced by psychological, social, biological, religious, spiritual, political, legal, economic, ethical, and historical issues.

Sexual development is considered a multidimensional process that is experienced more intensely by adolescents as they go through the changes of puberty, develop the capacity for intimacy, and experience sexual thoughts [2]. Adolescence is a complex stage of life that brings several challenges to health professionals. By traditional definition, it includes individuals from 10 to 19 years old. However, the definition has long posed a conundrum, and recently Sawyer at al. [3] suggested an extension to 10–24 years to correspond to a more current and updated concept. Regardless of age limits, it is a period of important biological growth, psychological development, and social role transitions.

The World Health Organization and other agencies consider sexual education as a priority within this age group due to sexual behavior implications, such as maternal mortality or sexually transmitted infections [4], and aspects such as gender-based violence and gender inequality. Since sexuality is an all-encompassing concept, there is no consensual definition of it. However, Goettsch [5] suggested a preliminary definition that includes: sexuality as an individual capacity, its experiential nature, body-oriented, and directed to genital excitation. The World Health Organization [1] gives a more comprehensive definition of sexuality that includes reproduction, sex, sexual orientation, gender identities and roles, pleasure, and intimacy, highlighting that not all of them are always experienced or expressed.

For many years now, schools have been considered privileged contexts for the implementation of health promotion and education, as they are built to foster personal and social development. Schools are the ideal place for building health-promoting communities, and are able to link children, families, and communities with other services (e.g., health services). The role of schools in health promotion is not a new phenomenon. In fact, historically, implementing health education interventions in schools has been recommended by several organizations and in several countries [6].

School-based education includes sexuality in its educational curricula, which is different from interventions, that can be implemented in a more/less structured way. Both approaches are used worldwide according to the context characteristics. School-based interventions are wildly recognized for their impact on adolescents’ sexual health [6]. Many sex education programs, especially those centered on risks associated with sexual activity, focus on knowledge as a prerequisite for adopting preventive behaviors. However, results from surveys such as the Health Behavior in School-Aged Children showed a trend of systematic reduction in terms of knowledge and protective behaviors [7]. Simultaneously, scientific evidence has shown that existing knowledge is not directly expressed in preventive practices, and that programs that include an ecological and participatory approach are more effective [8]. Thus, the focus of intervention programs has been expanding at different levels: from risk prevention to well-being, from directed and specific to individual to comprehensive and structural, and from traditional knowledge transference to innovative tools [9].

Interventions such as school policy changes, parent involvement, and work with local communities have been identified as effective for promoting sexual health [10]. In addition, the influence of contextual factors such as peers, parents, siblings, and schools have been examined and established by researchers [11]. As the field of intervention has expanded, it became evident that sexual education and promotion has a multidisciplinary and cross-sectoral nature. Interventions are broad, and are implemented by different professionals such as teachers, social workers, or psychologists, among others.

In this context, nurses are often involved in schools’ health promotion programs in general, but their role in these programs is not always clear or visible. Depending on the different realities, they can be in school-based health centers, or work in partnership with teachers or as part of multidisciplinary school health teams, and are often the only link between children and the health system [12]. They work directly with children and adolescents, but also collaborate in the training of teachers and other school personnel [13]. Additionally, annual preventive health examinations are seen as encounters in which screening adolescents for sexual and reproductive health is recommended [14]. Nurses work in a wide variety of settings (hospitals, health centers, schools), which gives them direct access to adolescent populations. In addition, nurses have an important role in promoting accessibility, inclusive communication, competent and guideline-based care, and confidentiality. Barriers to the implementation of health programs, identified by teachers, include lack of training and lack of time [8]. The integration of nurses in these programs represents an added value, and their unique combination of knowledge and specific skills [14] make them an essential and central element in these teams. A preliminary literature search revealed that the available studies on sexual education interventions are dispersed. Specific studies on nursing interventions in the school context also are scarce. The role of nursing and its unique contributions to this area remain less clear and less explored. Therefore, it is fundamental to firstly map the wide spectrum of interventions to promote adolescent sexuality, carried out in the school context. This can further contribute to a clearer identification of nursing’s role, and to the planning of interventions that are scientifically accurate, sustainable, and age- and culturally appropriate.

A scoping review was conducted according to the steps defined by Tricco et al. [15] that aimed to identify interventions that promote a healthy sexuality among school adolescents. This methodology proved to be advantageous, as it allowed identifying gaps in the literature, mapping the available evidence [16] that will support the planning of interventions to promote a healthy sexuality among adolescents in schools. The following research question was defined, according to PICo: which are the interventions that promote a healthy sexuality among school adolescents? (Population: adolescents; Interest: interventions to promote a healthy sexuality; Context: school).

## 2. Materials and Methods

The steps defined by Tricco et al. [15] for the scoping review were followed as detailed below.

Protocol and Registration. The protocol was drawn according to the Preferred Reporting Items for Systematic Reviews and Meta-Analyses extension for Scoping Reviews (PRISMA-ScR) and registered prospectively with the Open Science Framework on 18 June 2021 (https://osf.io/v97ek/?view_only=).

Eligibility criteria. Considering the scarcity of studies on specific nursing interventions, and keeping in mind the multidisciplinary scope of health promotion, for the purposes of this study, we selected studies focused on multidisciplinary interventions. Published articles on nurses, teachers, psychologists, and social care professionals integrated in work groups on sexuality in adolescents from school context were considered for analysis. Empirical studies with quantitative, qualitative, and mixed methods were included to maximize the coverage of evidence available. Peer-reviewed papers available in open access and full text and written in English, Spanish, and Portuguese were included. As to the publication date, no time frame was established. Manuscripts were excluded if studies were performed in specific contexts and specific health conditions (for example, hospitalization or adolescents with mental disorders). Letters to the editor, editorials, literature reviews, theoretical studies, protocols, methodological studies (for example, instrument validation/construction), blog articles, advertising, and opinion articles were excluded. Studies in which subjects were family members or health professionals that did not identify or suggest any interventions were also excluded.

Information sources. A three-step approach was used, as recommended in the literature [17]. The search was initially performed in two databases: Medical Literature Analysis and Retrieval System Online (MEDLINE) and Cumulative Index to Nursing and Allied Health Literature (CINAHL). Then, an analysis of the words contained in the titles and summaries was performed to understand the best terms to be used in the review. The purpose was to identify the keywords to be included in search equation.

In step two, after identifying the MeSH keywords to be used, research was conducted on the electronic platform EBSCOhost in the following databases: Cumulative Index to Nursing and Allied Health Literature (CINAHL) (complete); MEDLINE (complete); Nursing & Allied Health Collection (comprehensive); Cochrane Central Register of Controlled Trials; Cochrane Database of Systematic Reviews; Cochrane Methodology Register; Library, Information Science & Technology Abstracts (LISTA); and MedicLatina. Additionally, PubMed, SciELO, ScienceDirect, and Web of Science were also searched. For grey literature, we used Open Grey, MedNar, and WorldWideScience.org—The Global Science Gateway.

In step three, the list of references from the articles selected in step two were systematically searched to find additional relevant literature for this review.

Search. The research equation, designed with keywords and Boolean operators, was: ((Adolescen *) AND (Sexuality) AND (Nurs *)). An asterisk operator (*) was used, so that the database could identify variants of the original word. Research was performed in March 2021 by all the authors, working in groups of two (F.L. and V.A.; P.O. and M.F.). Considering inclusion criteria and fields available in databases, the sample initially obtained was limited, as shown in Table A1 (Appendix A).

Selection of sources of evidence. Articles were selected initially by title, and when it was not clear if the article fitted this review, the abstract was read. Duplicates were removed, and inclusion/exclusion criteria were applied. Reviewers in groups of two (F.L. and P.O.; V.A. and M.F.) screened the same publications to increase consistency. Any disagreements between reviewers were resolved through discussion with all reviewers until consensus was reached.

Data-charting process. The variables to be extracted were decided by all researchers, and a data-charting table was developed. The process was initially performed individually by each author. Then, working groups of two were formed (F.L. and P.O.; V.A. and M.F.) to compare the extracted data, resolve disagreements, and increase accuracy. In cases in which the articles contained insufficient information, the authors were contacted. The final extraction chart was discussed by all authors until reaching unanimity.

Data items. Data were extracted related to article characteristics such as reference, country, and methods (aim, population and sample, type of study). We also included the intervention implemented, as well as the main findings.

Critical appraisal of individual sources of evidence. To describe the quality of the selected articles (*n* = 56), studies were appraised by all authors. Divergent views regarding the critical appraisal were reviewed until consensus. The Hawker et al. [18] assessment tool, with a four-grade scale (1 = very poor; 2 = poor; 3 = fair; 4 = good) was used. The total score ranges between 9 and 36, and higher scores indicate higher quality. An article’s quality appraisal was centered on the following items: 1—abstract and title; 2—introduction and aims; 3—method and data; 4—sampling; 5—data analysis; 6—ethics and bias; 7—results; 8—transferability or generalizability; and 9—implications and usefulness.

Synthesis of results. Results were synthetized in a table that included all information extracted individually and approved by all authors. The data collected summarized studies and interventions that were identified in this review. Information was collected related to article reference, country where the study was applied, methods, intervention, and findings.

## 3. Results

**Figure 1:**
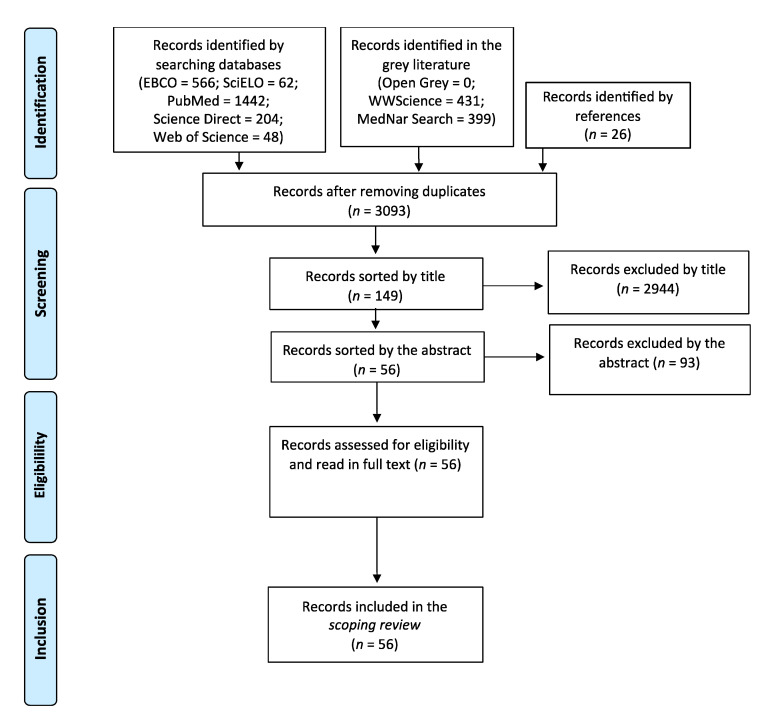
PRISMA flow chart of the study selection.

Selection of sources of evidence. A total of 149 articles were identified by title. From those, 93 were excluded by abstract. Articles excluded were mainly those that did not identify interventions. Manuscripts that presented only data concerning descriptive statistics for a diagnostic purpose in a particular context were also excluded. The process of study selection is summarized below in Figure 1 using a PRISMA flow chart.

Characteristics of sources of evidence. This review allowed the identification of a broad set of interventions to promote a healthy sexuality among adolescents in the school context. Through the International Classification of Health Interventions (ICHI), the World Health Organization [19] defines a health intervention as an act that has the purpose of assessing, improving, maintaining, promoting, or modifying health, functioning, or health conditions. These interventions can be performed for, with, or on behalf of a person or population. According to ICHI descriptors, interventions regarding sexuality can be mostly framed as interventions on body systems and function (ICHI code 1) or interventions on health-related behaviors (ICHI code 4). In this review, both types were retrieved from articles; however, interventions predominantly were oriented toward behaviors. On the other hand, it was not always clear which type of intervention was implemented; therefore, we categorized them as presented in Table A2 (Appendix A). The interventions were grouped by ascending order of frequency found in sample studies (*n* = 56).

Critical appraisal within sources of evidence. The results demonstrated that overall, the studies’ quality was high. Quality appraisal ranged from 18 to 35. Sampling, ethics, and bias, as well as transferability, were the main limitations of the studies (Table A3—Appendix A).

Results of individual sources of evidence. The results from each study selected in this scoping review are synthetized in Table A4 (Appendix A).

Synthesis of results. A total of 56 studies published between 1998 and 2021 were selected for final analysis. All studies had adolescents as subjects except for Barnes et al. [20], who studied nurses; and Valli and Cogo [21], whose study was related to school blogs on sexuality. Sample sizes ranged from 10 [22] to 11840 [23]. As to publication date, more than half of our article sample (*n* = 32) were published in the last 10 years. Regarding the country where studies were applied, we verified that there were studies from all around the world; however, three countries stood out: Brazil, with 16 articles; the USA, with 13 articles; and the UK, with 6 articles. Of the studies, 32 were of a quantitative nature, 18 used a qualitative approach, and 6 used mixed methods. Surveys were the most used form of collecting data (28), followed by combined techniques. These techniques included the use of surveys combined with interviews [24,25,26], focus groups [27,28], and an online environment [29]. A field diary and participating observation were also found as combined data collection techniques [22,30].

## 4. Discussion

Summary of evidence. The results indicated that adolescent sexuality is a relevant, current, and growing interest theme for health professionals, as we found articles from all around the world, and the great majority were recently published.

Evidence was mapped regarding the type of interventions performed in the school context, the factors that may influence their effectiveness, and a few considerations that were found on the multidisciplinary nature of school-based interventions.

Although studies that described an isolated intervention were found, mostly articles reported the findings of multiple interventional programs, such as SHARE [31,32,33] and Cuídate [34,35,36], or combined interventions, as stated above. The diversity of interventions found also reinforced the idea that adolescent health is still an evolving area [37] that requires a multidisciplinary approach.

Innovative approaches were also described, such as the use of blogs [21], Facebook [29], and online games [27,38], as they seemed to have greater acceptance by adolescents. As technologies continue to expand, online resources are becoming progressively relevant and used by adolescents, which raises concerns regarding vulnerability, but also presents opportunities for important impacts on healthy learning [39].

Interestingly, mobile phone intervention was found in six studies. Given the access and amount of time spent by adolescents with these devices, this intervention seems particularly suitable for the adolescent population. As an intervention that uses existing resources, mobile phone intervention is cost effective and sustainable, with the ability to reach many adolescents even in low-income countries. Furthermore, there is evidence that the use of mobile text messages may lead to improved adolescent sexual and reproductive health [40].

Interventions delivered through school-based health centers (SBHCs) are described in two studies. The existence of SBHCs has proven to have an impact on adolescents regarding satisfaction with their health and adherence to health-promoting behaviors [41]. Not all countries have SBHCs, since they are not part of their health and education systems. Nevertheless, policy makers should consider including this strategy in their agendas, as it is a more comprehensive and integrated approach. It has proven to raise adolescents’ access to health care services, overcoming identified barriers such as charges, transportation, accessibility, availability, and privacy concerns [23].

Sex education sessions, group discussions, and workshops can be framed as the more traditional interventions. They can be implemented alone, simultaneously, integrated in a broader program, or used combined with other interventions. The studies that addressed them were mostly descriptive, with few considerations of their effectiveness as isolated interventions. With respect to sex education sessions, nurses often have a prominent role, particularly school nurses, as they have a broader knowledge of health-related issues. Brewin et al. [42] reported barriers related to privacy, time, confidentiality, and fear of conflict.

Peer education was only mentioned in four studies. This is an intervention that creates greater expectations around its impact on adolescent sexual behavior because, when performed and conveyed by peers with a similar age or status, it becomes more appealing and credible [43]. However, no study could demonstrate peer education effectiveness by itself. Stephenson et al. [43] argued that it should be considered as part of a broader program.

Concerning interventions’ effectiveness, some studies did not assess interventions’ impact on adolescent behavior or knowledge, only describing the implementation experience in the school context. Even so, a more comprehensive approach that includes multiple types of interventions seems to be more effective in promoting positive changes in sexual behavior [44]. These findings were aligned with the multidimensional scope of adolescent sexuality.

In general, the articles evaluating the effectiveness of programs demonstrated a very low sustainability in the modification of risk behaviors [31,36,43,45,46] of young people and adolescents. Of the articles included, only one showed maintenance of the positive effects in terms of reduced pregnancies, delayed sexual debut, and intentions to use condoms. Again, it described a more comprehensive approach: a training intervention for children’s social competence, classroom management and instruction, and parenting practices without interventions specifically aimed at sexuality [47].

In addition, interventions focused on psycho-affective and socio-emotional skills showed a greater impact on long-term behavioral changes. These findings were in line with the growing interest in socio-emotional skills learning (SEL) that, especially since the beginning of this century, has shown a positive impact on school success, well-being, and health of the participants [48], and which are at the base of health-promotion programs in schools such as the Collaborative for Academic, Social, and Emotional Learning (CASEL) (Chicago, IL, USA) or the Schools for Health in Europe Network Foundation (SHE) (Haderslev, Denmark).

Furthermore, we believe that the fact that programs are mostly implemented in local and regional contexts and only in a specific period of time limits their long-term effectiveness. Continuity seems to be a key factor in maintaining the change in adolescent behavior with regard to their sexuality.

The fact that so many studies and so many different interventions were found, with little evidence of their effectiveness, may also signify a difficulty in officially instituting and operationalizing them in the long term.

It is worth noting the difficulties in categorizing the domains of sexuality and standardizing the language regarding interventions. Despite the evident effort of the scientific community, there is still no universal consensus on them. Thus, there seems to be agreement on the multidisciplinary nature of interventions that promote adolescent sexuality.

As to limitations, we must consider the possibility of having excluded or missed some relevant studies due to the databases used, and the exclusion of studies written in languages other than English, Spanish, or Portuguese.

## 5. Conclusions

As previously mentioned, nurses are often involved in schools’ health-promotion programs in general, but their role in these programs is not always clear or visible. This research did not allow us to draw conclusions about this topic. Nonetheless, it is interesting to note that most studies had nurses or nursing professors as their first author, although they did not focus exclusively on the role of nurses/nursing care. Again, this can be explained by the multidisciplinary nature of the interventions.

Finding which interventions worked best to promote a healthy sexuality among school adolescents was challenging. As the studies were carried out in very different contexts, and there are few long-term evaluations of the implemented interventions, it was not possible to make considerations about their effectiveness.

This review allowed the identification of interventions implemented in schools to promote adolescents’ healthy sexuality; namely, event history calendar, group discussion, interventions delivered through SBHC, peer education, online intervention, mobile phone intervention, combined interventions, workshops, sex education sessions, and multiple interventional programs. These findings were in line with the multidimensional scope of adolescent sexuality. The studies found were recent and were published all around the world, which sustained the idea that this is a relevant and evolving theme. 

Although most authors were nursing professionals or nursing students, the particular role of nurses on the multidisciplinary team was not explored. This is clearly a gap in the evidence that requires further investigation. However, this review gave a clear picture of the interventions that can be implemented to promote adolescent sexuality. The effectiveness of those strategies should be further explored. Decision makers should integrate these strategies in their agendas and use them as collaboration measures between the health and education sectors.

## Appendix A

**Table A1:** Platforms and limiters used in search strategy.

Platform	Limiters
EBSCOhost	Adolescen * AND Sexuality AND Nurs * Portuguese, Spanish, or English language full text available
PubMed	Adolescen * AND Sexuality AND nurs * Portuguese, Spanish, or English language Full text available
SciELO	Adolescente OR adolescência AND sexualidade AND enfermagem
ScienceDirect	(Adolescent OR Adolescence) AND Sexuality AND (nursing OR nurse) Open access
Web of Science	Adolescen * AND Sexuality AND nurs * Portuguese, Spanish, or English language Full text available
Open Grey	Adolescen * AND Sexuality AND nurs *
MedNar	Adolescen * AND Sexuality AND nurs * Full text available
WorldWideScience	(Adolescent OR Adolescence) AND Sexuality AND (nursing OR nurse) Full text available

* Placing an asterisk is a strategy used in serach so that all variants of words are considered.

**Table A2:** Synthesis of interventions retrieved from studies.

Type of Intervention	Author, Year [49,50,51,52,53,54,55,56,57,58,59,60,61,62,63,64,65,66,67,68,69,70,71,72,73,74,75,76,77,78,79,80,81,82]
Event history calendar	(Martyn et al., 2012)
Group discussion	(Beserra et al., 2008; Fonseca et al., 2010)
Interventions delivered through School-Based Health Centers	(Bersamin et al., 2018; Denny et al., 2012)
Peer education	(Hatami et al., 2015; Okanlawon and Asuzu, 2013; Stephenson et al., 2008, 2004)
Online intervention	(Aragão et al., 2018; Castillo-Arcos et al., 2016; Enah et al., 2015; Souza et al., 2017; Valli and Cogo, 2013)
Mobile phone intervention	(Alhassan et al., 2019; Cornelius et al., 2019, 2012; French et al., 2016; Hickman and Schaar, 2018; Rokicki et al., 2017) [49]
Combined interventions	(Aventin et al., 2015; Barnes et al., 2004; Beserra et al., 2017; Gallegos et al., 2008; Hirvonen et al., 2021; Madeni et al., 2011; Oliveira et al., 2016)
Workshops	(Amaral and Fonseca, 2006; Beserra et al., 2006; Camargo and Ferrari, 2009; Carvalho et al., 2005; Freitas and Dias, 2010; Gubert et al., 2009; Levandowski and Schmidt, 2010; Santos et al., 2017; Soares et al., 2008) [50]
Sex education sessions	(Dunn et al., 1998; Elliott et al., 2013; Golbasi and Taskin, 2009; Grandahl et al., 2016; Lieberman et al., 2000; Moodi et al., 2013; Rani et al., 2016; Serowoky et al., 2015; Walker et al., 2006; Yakubu et al., 2019)
Multiple interventions program	(Henderson et al., 2007; Jemmott III et al., 1998; Jemmott et al., 2015; Lonczak et al., 2002; Richards et al., 2019; Siegel et al., 1998; Tucker et al., 2007; Villarruel et al., 2010, 2006; Wight et al., 2002)

**Table A3:** Critical appraisal of studies included in the scoping review.

Study	Abstract and Title	Introduction and Aims	Method and Data	Sampling	Data Analysis	Ethics and Bias	Results	Transferability or Generalizability	Implications and Usefulness	TOTAL
[58]	4	3	4	4	4	4	4	4	4	35
[59]	4	4	4	4	4	3	4	4	4	35
[24]	4	4	4	4	4	3	4	4	4	35
[49]	4	3	4	4	4	3	4	4	4	34
[47]	4	4	4	4	4	3	4	4	3	34
[74]	4	3	4	4	4	3	4	4	4	34
[43]	4	3	4	4	4	3	4	4	4	34
[51]	4	4	4	3	4	2	4	4	4	33
[23]	4	4	3	4	4	2	4	4	4	33
[27]	4	3	4	4	4	2	4	4	4	33
[45]	4	3	4	4	4	2	4	4	4	33
[70]	4	3	4	4	4	3	4	4	3	33
[80]	4	3	4	4	4	3	4	4	3	33
[33]	4	4	4	4	4	1	4	4	4	33
[82]	4	3	4	4	4	3	4	4	3	33
[64]	4	4	4	3	3	3	4	3	4	32
[65]	4	4	4	4	4	3	4	3	2	32
[31]	4	2	4	4	4	2	4	4	4	32
[72]	4	4	4	3	4	3	4	3	3	32
[77]	4	4	3	3	4	3	4	3	4	32
[54]	4	3	4	3	4	3	4	3	3	31
[57]	4	3	4	3	4	3	4	3	3	31
[46]	4	3	4	3	4	1	4	4	4	31
[34]	4	4	4	3	4	1	4	3	4	31
[79]	4	3	4	3	4	3	4	3	3	31
[21]	4	3	4	3	4	3	3	4	3	31
[35]	4	3	4	3	4	3	4	3	3	31
[81]	4	2	3	4	4	2	4	4	4	31
[61]	4	3	3	3	4	2	4	3	4	30
[68]	4	3	4	3	4	3	4	3	2	30
[69]	3	3	4	3	4	3	4	3	3	30
[25]	4	3	4	3	4	2	4	3	3	30
[30]	3	3	4	2	4	3	4	3	4	30
[32]	4	3	3	3	4	2	4	3	4	30
[29]	4	3	4	2	4	3	4	2	3	29
[22]	3	3	4	2	4	3	4	3	3	29
[56]	4	3	3	3	4	3	3	3	3	29
[36]	4	2	3	3	4	3	4	3	3	29
[26]	4	3	4	3	4	3	4	3	4	28
[75]	4	3	4	2	3	3	3	3	3	28
[53]	4	2	3	2	3	3	4	2	4	27
[66]	4	4	3	2	3	2	4	2	3	27
[38]	4	4	3	2	3	3	3	2	3	27
[20]	3	3	4	3	3	2	3	3	2	26
[76]	3	3	3	3	4	2	4	2	2	26
[55]	3	3	3	3	3	2	3	3	2	25
[62]	4	3	3	2	3	2	3	3	2	25
[73]	3	3	3	3	3	1	3	3	3	25
[28]	4	3	2	2	3	2	4	2	3	25
[63]	3	3	3	2	3	3	3	2	2	24
[67]	3	2	3	2	4	2	4	2	2	24
[50]	3	3	3	2	2	3	3	2	2	23
[52]	3	3	3	2	2	2	3	2	3	23
[60]	3	3	2	3	2	1	4	3	2	23
[71]	2	3	3	2	2	2	3	2	2	21
[78]	3	3	1	2	2	1	2	2	2	18

4 = Good, 3 = Fair, 2 = Poor, 1 = Very poor.

**Table A4:** Study characteristics.

Study	Country	Aim	Type of Study	Population and Sample	Interventions	Key Findings
(Hirvonen et al., 2021)	UK	To understand the perceptions of adolescent students regarding the use of Facebook social media in sexual and reproductive health learning.	Mixed methods	1413 teenagers (14 to 16 years)	Combined interventions: peers’ intervention and use of private Facebook groups	Social media groups formed around peer supporters’ existing friendship networks hold potential for diffusing messages in peer-based sexual health interventions.
(Alhassan et al., 2019)	Ghana	To assess mobile phone usage among adolescents and young adult populations pursuing tertiary education and their use of these technologies in the education and prevention of STI.	Quantitative	250 teenagers (18 to 24 years)	Mobile phone interventions	Future mobile phone programs should be considered for STI education and prevention, as they were found to be more comfortable than traditional messaging or phone calls.
(Cornelius et al., 2019)	USA	To examine adolescents in the USA and Botswana, their mobile phone and social media usage, and their perceptions of safer-sex interventions delivered via social media.	Qualitative	28 teenagers (13 to 18 years)	Mobile phone interventions	Findings provided a starting point for researchers interested in developing a social media intervention with global implications for sexual health promotion.
(Golbasi and Taskin, 2009)	Turkey	To evaluate the effectiveness of school-based reproductive health education for adolescent girls on the reproductive knowledge level of the girls.	Quasi- experimental	189 adolescents (age not mentioned)	Sex education sessions	The school-based program was conducted by nursing educators, and the program was effective in increasing the students’ levels of knowledge on reproductive health.
(Richards et al., 2019)	Dominican Republic	To evaluate the effectiveness of MAMI’s CSEP in changing knowledge of STIs and pregnancy.	Mixed methods	600 students aged 11–25 years old	Multiple interventional program (comprehensive sexual education program: education sessions, interactive activities, visual aids)	The MAMI’s CSEP improved knowledge of STIs and pregnancy and attitudes toward risky sexual behavior among program recipients.
(Yakubu et al., 2019)	Ghana	To assess an educational intervention program on knowledge, attitude, and behavior toward pregnancy prevention based on the HBM amongst adolescent girls in Northern Ghana.	RCT	363 adolescents (13 to 19 years).	Sex education sessions	Educational intervention, which was guided by HBM, significantly improved sexual abstinence and the knowledge of adolescents on pregnancy prevention.
(Aragão et al., 2018)	Brazil	To understand the perceptions of adolescent students regarding the use of Facebook social media in sexual and reproductive health learning.	Qualitative	96 adolescents (mean age 15 years)	Online interventions (Facebook)	Virtual spaces on the Internet offer potential for the production of health care, especially among adolescents, as it contributes to the sexual and reproductive health education in an interactive, playful, and practical way.
(Bersamin et al., 2018)	USA	To investigate the associations between SBHCs and sexual behavior and contraceptive use among 11th graders.	Quantitative	11840 adolescents (average age 16.6)	Interventions delivered through SBHCs	Exposure to SBHCs in general, and availability of specific reproductive health services, may be an effective strategy to support healthy sexual behaviors among youth.
(Hickman and Schaar, 2018)	USA	To develop and evaluate adolescent satisfaction with a text-messaging educational intervention to promote healthy behaviors, reduce the incidence of unhealthy behaviors, and prevent high-risk behaviors.	Mixed methods	202 adolescents (14 to 18 years)	Mobile phone interventions	Text messaging is a good way to educate adolescents and promote healthy habits, as it shows a high rate of intended behavioral change by adolescents.
(Beserra et al., 2017)	Brazil	To analyze the perception of adolescents about the life activity “express sexuality”.	Qualitative	25 teenagers (15 to 18 years)	Combined interventions: video projection followed by discussion and clarification of doubts	The use of videos followed by discussion is a valid and useful strategy to help adolescents in expressing their sexuality.
(Rokicki et al., 2017)	Ghana	To evaluate whether text-messaging programs can improve reproductive health among adolescent girls in low- and middle-income countries.	RCT	756 adolescents (14 to 24 years)	Mobile phone interventions	Text-messaging programs can lead to large improvements in reproductive health knowledge, and have the potential to lower pregnancy risk for sexually active adolescent girls.
(Santos et al., 2017)	Brazil	To report the experience of conducting a workshop with teenagers about STIs.	Qualitative	34 adolescents (average age, 18 years old)	Workshops	Group educational experiences provide adolescents with the opportunity to build shared knowledge, and professionals learn about adolescents’ doubts, and therefore plan new health education meetings.
(Souza et al., 2017)	Brazil	To describe the online game and reflect on its theoretical– methodological basis.	Qualitative	60 adolescents aged 15–18 years	Online game (Straight Talk)	Conceived as a pedagogic device, the online game has the capacity to implicate the adolescent in problem-based situations and allows the invention of other forms to deal with sexuality without the demand of support from a teacher.
(Castillo-Arcos et al., 2016)	Mexico	To evaluate the effect of an internet-based intervention to reduce sexual risk behaviors and increase resilience to sexual risk behaviors among Mexican adolescents.	Quasi- experimental	193 adolescents (14 to 17 years)	Online interventions (sex education sessions)	The intervention improved self-reported resilience to risky sexual behaviors, though not with a reduction in those behaviors.
(French et al., 2016)	UK	To explore young people’s views of and experiences with a mobile phone text-messaging intervention to promote safer-sex behavior.	Qualitative	20 adolescents (16 to 24 years)	Mobile phone interventions	The intervention increased knowledge, confidence, and safer-sex behaviors.
(Grandahl et al., 2016)	Sweden	To improve primary prevention of HPV by promoting vaccination and increased condom use among upper secondary schools.	RCT	741 adolescents (aged 16 years)	Sex education sessions (face-to-face structured interventions)	Face-to-face education delivery by health care providers, such as school nurses, is a highly feasible and effective way to increase adolescents’ beliefs and behavior toward primary prevention of HPV, regardless of socioeconomic status, ethnicity, or cultural background.
(Oliveira et al., 2016)	Brazil	To analyze the limits and the potentialities of the Papo Reto game, for construction of knowledge in the field of sexuality with adolescents.	Qualitative	23 adolescents (15 to 18 years)	Combined interventions (virtual game, workshops)	The game can be used as a pedagogic device for dealing with the subject of sexuality in adolescents. The results confirmed the potentiality of the contents for dealing with the complexity of reality from the point of view of gender.
(Rani et al., 2016)	India	To compare the knowledge and attitude regarding pubertal changes among pre-adolescent girls before and after the pubertal preparedness program	Quasi- experimental	104 pre- adolescent (12–14 years)	Sex education sessions	Pubertal preparedness programs and FAQs reinforcement sessions are effective in enhancing knowledge and developing a favorable attitude among pre-adolescent girls.
(Aventin et al., 2015)	Ireland	To design, develop, and optimize an educational intervention about young men and unintended teenage pregnancy based around an interactive film.	Mixed methods	360 adolescents (14 to 17 years)	Combined interventions: interactive film-based interventions (If I were Jack) with group discussion	The model of intervention reported in this paper was presented not as an ideal, but as an exemplar that other researchers might utilize, modify, and improve.
(Enah et al., 2015)	USA	To assess the acceptability and relevance of a web-based HIV prevention game for African American rural adolescents.	Mixed methods	42 adolescents (12 to 16 years)	Online interventions (game: Fast Car)	Using games for HIV prevention was found to be appealing and acceptable, but it was not found to be the best approach to HIV prevention with the target population.
(Hatami et al., 2015)	Iran	To evaluate the effect of organizing interactions using peer education in schools on the knowledge and attitude toward sexual health.	Quantitative	282 teenagers (14 to 18 years)	Peer education	The use of peer education in schools informally could enhance the knowledge and approach toward aspects of physical health, sexual behaviors, and social and mental changes among female adolescents.
(Jemmott et al., 2015)	2015 South Africa	To test the effect of an HIV/STI risk-reduction intervention.	RCT	1057 adolescents 9–18 years old	Sex education sessions	The HIV/STI risk-reduction intervention reduced unprotected intercourse and caused positive changes on theoretical constructs. Theory-based behavioral interventions with early adolescents can have long-lived effects in the context of a generalized severe HIV epidemic.
(Serowoky et al., 2015)	USA	To plan, implement, and evaluate a sustainable model of sexual health group programming (Cuídate) in a high school with a large Latino student population.	Quasi- experimental	24 adolescents (13 to 18 years)	Multiple interventional program (Cuídate)	The intervention showed significant increases in STI or HIV knowledge, self-efficacy, and intention to use condoms. It can be sustained in a school-based health center with results of efficacy.
(Elliott et al., 2013)	UK	To assess the effectiveness of the Scottish government’s National Sexual Health Demonstration Project (HR2).	Quasi- experimental	5283 pupils aged 15–16 years	Sex education sessions	Combining sex education and sexual health services has a limited impact on young people’s sexual health.
(Moodi et al., 2013)	Iran	To evaluate the effect of an educational program for puberty health on improving intermediate and high school female students’ knowledge in Birjand, Iran.	Quasi- experimental	302 female students (mean age 12.9)	Sex education sessions	Performing educational programs during puberty has a crucial role in young girls’ knowledge increase.
(Okanlawon and Asuzu, 2013)	Nigeria	To involve adolescents in school-based health-promotion activities that would improve their perception of risk in sexual behavior.	Quasi- experimental	519 adolescents	Peer education	Adolescents’ active participation in health-promotion activities should be encouraged, as it improves the perception of risk in sexual behavior among adolescents.
(Valli and Cogo, 2013)	Brazil	To analyze the structure of school blogs on sexuality and their utilization by adolescents.	Quantitative	11 blogs about sexuality	Online interventions (blog)	The blog is a virtual interaction tool common among adolescents that allows the adolescent to establish relationships with other teens interested in the topic, decreasing feelings of doubt, isolation, and shyness.
(Cornelius et al., 2012)	USA	To understand adolescents’ perceptions of mobile cell phone text-messaging-enhanced and mobile cell phone-based HIV- prevention interventions.	Qualitative	11 teenagers (13 to 18 years)	Mobile phone interventions	The messages increased participants’ HIV awareness and knowledge.
(Denny et al., 2012)	New Zealand	To determine the association between availability and quality of school health services and reproductive health outcomes among sexually active students.	Quantitative	2745 adolescents (13 to 17 years)	Interventions delivered through SBHCs	Health services may be able to lower the incidence of pregnancy by providing access to comprehensive health services, including contraceptive care that is easily available and appropriate for the student population.
(Martyn et al., 2012)	USA	To explore the effects of an event history calendar approach on adolescent sexual risk communication and sexual activity.	Mixed methods	30 adolescents (15 to 19 years)	Event history calendar	School nurses could use the event history calendar approach to improve adolescent communication on sexual risks and tailoring of interventions.
(Madeni et al., 2011)	Tanzania	To evaluate a reproductive health awareness program for the improvement of reproductive health for unmarried adolescent girls and boys in urban Tanzania.	Quasi- experimental	305 adolescents (11 to 16 years)	Combined interventions (sex education sessions and group discussions)	The reproductive health program improved the students’ knowledge and behavior about sexuality and decision making after the program for both girls and boys. Their attitudes were not likely to change based on the educational intervention.
(Fonseca et al., 2010)	Brazil	To find the perception of adolescents on the sexual orientation actions in a public school and to identify the actions’ fragilities and potentials.	Qualitative	15 adolescents (15 to 17 years)	Group discussions	Participative methodology promotes a welcoming and productive work climate and provides larger involvement and learning.
(Freitas and Dias, 2010)	Brazil	To understand teenagers’ perceptions about the development of their sexuality.	Qualitative	12 teenagers (11 to 19 years)	Workshops	Teenagers’ perceptions about their sexuality emerged from the debates and shared knowledge during the workshops.
(Levandowski and Schmidt, 2010)	Brazil	To enable the exchange of experiences and reflection about sexuality-related actions and choices.	Qualitative	270 adolescents (12 to 15 years)	Workshops	The intervention reduced psychosocial risk factors, providing a healthy development.
(Villarruel et al., 2010)	Mexico	To examine the effectiveness of a safer-sex program (Cuídate) on sexual behavior, use of condoms, and use of other contraceptives among Mexican youth 48 months after the intervention.	RCT	708 adolescents (mean age 19.22)	Multiple interventional program (Cuídate)	Results demonstrated the efficacy of Cuídate among Mexican adolescents. Future research, policy, and practice efforts should be directed at sustaining safe-sex practices across adolescents’ developmental and relationship trajectories.
(Camargo and Ferrari, 2009)	Brazil	To analyze the knowledge of adolescents on sexuality, contraceptive methods, pregnancy, and STDs/AIDS before and after prevention workshops.	Qualitative	117 adolescents (14 to 16 years)	Workshops	There is a need for systematic work, in the medium and long term, in schools regarding adolescent sexuality, as there was no increase in knowledge about STD transmission methods.
(Gubert et al., 2009)	Brazil	To address the use of educational technology as a strategy for health education among the teenagers in the school.	Qualitative	30 teenagers (14 to 18 years)	Workshops	Nursing professionals should produce/ readjust new technologies that support the educational process in health education, valuing the skills and aspirations of adolescents, going beyond traditional health education activities based on specific actions and that do not recognize the real needs.
(Beserra et al., 2008)	Brazil	To investigate the adolescents’ sexuality from the educative action of a nurse in the prevention of STDs.	Qualitative	10 adolescents (14 to 16 years)	Group discussions	The intervention allowed adolescents to explore and discuss many subjects that involved their sexuality, and it was also a moment to take actions on health education with the objective to prevent risks.
(Gallegos et al., 2008)	Mexico	To test the efficacy of a behavioral intervention designed to decrease risk of sexual behaviors for HIV/AIDS and unplanned pregnancies in Mexican adolescents.	RCT	832 adolescents, age 14–17	Combined interventions: sex education sessions and interactive games	The behavioral intervention represented an important effort, and was effective in promoting safe sexual behaviors among Mexican adolescents.
(Soares et al., 2008)	Brazil	To understand how adolescents live and exercise their sexuality.	Qualitative	350 adolescents (15 to 19 years)	Workshops	The workshops favored the discussion of attitude changes in the adolescents through the information, reflection, and expression of ideas and feelings, representing a process to be complemented by the family, school, and local social politics.
(Henderson et al., 2007)	Scotland	To assess the impact of a theoretically based sex education program (SHARE) delivered by teachers compared with conventional education in terms of conceptions and terminations registered by the NHS.	RCT	4196 female (mean age 20)	Multiple intervention program (SHARE)	Enhanced teacher-led school sex education (SHARE) improved knowledge and reduced regret, but did not reduce conceptions or terminations compared with conventional sex education.
(Tucker et al., 2007)	2006 UK-Lothian	To test for improved outcomes for the new Lothian Healthy Respect’s SHARE on teenage sexual behavior outcomes in the Lothian region.	Quasi- experimental	4381 secondary school pupils (average age 14.6 years)	Multiple intervention program (SHARE)	The findings demonstrated limited impact on sexual health behavior outcomes and raised questions about the likely and achievable sexual health gains for teenagers from school-based interventions.
(Amaral and Fonseca, 2006)	Brazil	To understand adolescents’ social representations on sexual initiation concerning gender.	Qualitative	16 adolescents (11 to 16 years)	Workshops	The strategy allowed the understanding of the social representations of teenagers about sexual initiation, being of great importance for planning the work developed with teenagers, supporting debates and reflections on the experience of healthy and responsible sexuality by young people.
(Beserra et al., 2006)	Brazil	To describe an experience to promote health and prevent STDs among teenagers.	Qualitative	28 adolescents (13 to 16 years)	Workshops	The strategy was effective at promoting teenagers’ adoption of preventative measures.
(Villarruel et al., 2006)	USA	To test the efficacy of a prevention intervention to reduce sexual risk behavior among Latino adolescents.	RCT	553 adolescents, aged 13 to 18 years	Multiple interventional program (Cuídate)	Results provided evidence for efficacy for HIV prevention in decreasing sexual activity and increasing condom use among Latino adolescents.
(Walker et al., 2006)	Mexico	To assess effects on condom use and other sexual behavior of an HIV prevention program at school that promotes the use of condoms with and without emergency contraception.	RCT	First-year high school students (*n* = 10954) (16–17 years)	Sex education sessions	A rigorously designed, implemented, and evaluated HIV education course based in public high schools did not reduce risk behavior, so such courses need to be redesigned and evaluated.
(Carvalho et al., 2005)	Brazil	To determine how the intervention in sexual guidance was experienced by adolescents.	Qualitative	13 adolescents (13 to 15 years)	Workshops	The analysis demonstrated a reconstruction /redefinition of meaning for the ideas related to sexuality, to gender, and to the wider social context.
(Barnes et al., 2004)	Australia	To evaluate the impact of changes in the health system and services on the roles and responsibilities of child health nurses and to identify professional development needs.	Qualitative	10 nurses	Combined interventions: health education and health information displays.	The school-based youth health nurse program provides nurses with a new, challenging, autonomous role within the school environment, and the opportunity to expand their role to incorporate all aspects of the health-promoting schools’ framework.
(Stephenson et al., 2004)	UK	To examine the effectiveness of one form of peer-led sex education.	RCT	8000 students (13 to 14 years)	Peer education	Peer-led sex education was effective in some ways, but broader strategies are needed to improve young people’s sexual health. The role of single-sex sessions should be further investigated.
(Lonczak et al., 2002)	USA	To examine the long-term effects of the full SSDP intervention on sexual behavior and associated outcomes assessed at age 21 years.	Non- randomized controlled trial	349 former fifth-grade students (aged 21 years)	Multiple interventional program (SSDP)	A theory-based social development program that promotes academic success, social competence, and bonding to school during the elementary grades can prevent risky sexual practices and adverse health consequences in early adulthood.
(Wight et al., 2002)	Scotland	To determine whether a theoretically based sex education program for adolescents (SHARE) delivered by teachers reduced unsafe sexual intercourse compared with current practice.	RCT	8430 pupils aged 13–15 years	Multiple intervention program (SHARE)	Compared with conventional sex education, this specially designed intervention did not reduce sexual risk-taking in adolescents.
(Lieberman et al., 2000)	USA	To assess the impact of an abstinence-based model for sexual education.	Quantitative	312 students (mean age 12.9)	Multiple intervention program (IMPPACT)	A small-group abstinence-based intervention can have some impact on adolescents’ attitudes and relationships (particularly with their parents).
(Dunn et al., 1998)	Canada	To evaluate a school-based HIV prevention intervention in adolescents.	Quasi- experimental	160 adolescents (14 to 15 years)	Sex education sessions	School-based interventions can improve adolescents’ short-term HIV/AIDS prevention knowledge, attitudes, self-efficacy, and behavioral intentions.
(Jemmott III et al., 1998)	USA	To evaluate the effects of abstinence and safer-sex HIV risk-reduction interventions on young inner-city African American adolescent’s HIV sexual risk behaviors when implemented by adult facilitators as compared with peer cofacilitators.	RCT	659 adolescents (mean age 11.8)	Sex education sessions	Both abstinence and safer-sex interventions can reduce HIV sexual risk behaviors, but safer-sex interventions may have longer-lasting effects, and may be especially effective with sexually experienced adolescents.
(Siegel et al., 1998)	USA	To determine the short-term effect of a middle and high school–based AIDS and sexuality intervention (RAPP) on knowledge, self-efficacy, and behavior intention.	Non- randomized controlled trial	Middle and high school students (*n* = 3635)	Multiple interventional program (RAPP): games, role playing, take-home exercises	At short-term follow-up, the RAPP intervention had a powerful effect on knowledge for all students and a moderate effect on sexual self-efficacy and safe behavior intention, particularly for high school students.
(Stephenson et al., 2008)	UK	To assess the long-term effects of a peer-led sex education program.	RCT	9000 students (13–14 years)	Peer education	Compared with conventional school sex education at age 13–14 y, this form of peer-led sex education was not associated with a change in teenage abortions, but may have led to fewer teenage births, and was popular with pupils. It merits consideration within broader teenage-pregnancy- prevention strategies.
